# The Use of Snuff

**Published:** 1859-03

**Authors:** 


### THE USE OF SNUFF.

Mrs. Mary E. Bryan, the gifted Editress of the "Ladies Department" of the *Crusader*, (Atlanta, Ga.,) thus forcibly discourses in reference to a most disgusting and destructive habit:

"Be it remembered, then, that our "voice is for war" against Tobacco in general, and snuff in particular, and that we have pitted our "gray goose quill" against all the snuff brushes in the land. The battle is not *always* to the strongest.

But seriously, this evil of snuff-using is a great and extended one, and calls for more eloquence than we can command, to inveigh against it. The practice is a *hundred times* more general than is believed, or even suspected, and if the foe is more insiduous and less swift than Alcohol, it is hardly less destructive.

We dare not tell you how many among our lady acquaintances are secretly slaves to this pernicious habit. We know daughters, whose snuff bottles are concealed in their rooms, where they use the contents constantly, without the knowledge even of their parents. We have seen, at boarding schools, girls go into hysterics when deprived forai day or two of their snuff, and *borrow tobacco from the servants*, unt l they could obtain their usual stimulant of Maccaboy; and we are well acquainted with three sisters—beautiful young girls, were it not for the sallow hue tarnishing their complexions—who are at present under medical treatment for derangement of the nervous system and digestive organs, arising from their *constant* use of snuff. Their physician has assured them that this was the cause of the disease, thus blighting their young lives, and that medicine must be in vain as long as the practice was continued, and still, they cling to their snuff bottles as persistently as the toper to his demijohn. And this when they *know* that this vile poison nourishes the worm of the disease at the root of life, silently, slowly, but surely destroying it ere its prime; for aside from the disgusting filthiness of this habit, the constant drain of the salivary glands, produced by frequent spitting and the narcotic poison of the weed itself, throw the delicately balanced system out of order, and bring a train of diseases to render life insupportably burdensome."

